# PI3K p110γ Deletion Attenuates Murine Atherosclerosis by Reducing Macrophage Proliferation but Not Polarization or Apoptosis in Lesions

**DOI:** 10.1371/journal.pone.0072674

**Published:** 2013-08-22

**Authors:** Teresa M. Zotes, Cristina F. Arias, José J. Fuster, Roberto Spada, Sonia Pérez-Yagüe, Emilio Hirsch, Matthias Wymann, Ana C. Carrera, Vicente Andrés, Domingo F. Barber

**Affiliations:** 1 Department of Immunology and Oncology, Centro Nacional de Biotecnología-Consejo Superior de Investigaciones Científicas (CNB-CSIC), Madrid, Spain; 2 Department of Epidemiology, Atherothrombosis and Imaging, Centro Nacional de Investigaciones Cardiovasculares (CNIC), Madrid, Spain; 3 Department of Genetics, Biology and Biochemistry, Center for Molecular Biotechnology-University of Torino, Torino, Italy; 4 Department of Clinical and Biological Sciences, Institute of Biochemistry and Genetics- University of Basel, Basel, Switzerland; University of Amsterdam Academic Medical Center, Netherlands

## Abstract

Atherosclerosis is an inflammatory disease regulated by infiltrating monocytes and T cells, among other cell types. Macrophage recruitment to atherosclerotic lesions is controlled by monocyte infiltration into plaques. Once in the lesion, macrophage proliferation *in situ*, apoptosis, and differentiation to an inflammatory (M1) or anti-inflammatory phenotype (M2) are involved in progression to advanced atherosclerotic lesions. We studied the role of phosphoinositol-3-kinase (PI3K) p110γ in the regulation of *in situ* apoptosis, macrophage proliferation and polarization towards M1 or M2 phenotypes in atherosclerotic lesions. We analyzed atherosclerosis development in LDLR^−/−^p110γ^+/−^ and LDLR^−/−^p110γ^−/−^ mice, and performed expression and functional assays in tissues and primary cells from these and from p110γ^+/−^ and p110γ^−/−^ mice. Lack of p110γ in LDLR^−/−^ mice reduces the atherosclerosis burden. Atherosclerotic lesions in fat-fed LDLR^−/−^p110γ^−/−^ mice were smaller than in LDLR^−/−^p110γ^+/−^ controls, which coincided with decreased macrophage proliferation in LDLR^−/−^p110γ^−/−^ mouse lesions. This proliferation defect was also observed in p110γ^−/−^ bone marrow-derived macrophages (BMM) stimulated with macrophage colony-stimulating factor (M-CSF), and was associated with higher intracellular cyclic adenosine monophosphate (cAMP) levels. In contrast, T cell proliferation was unaffected in LDLR^−/−^p110γ^−/−^ mice. Moreover, p110γ deficiency did not affect macrophage polarization towards the M1 or M2 phenotypes or apoptosis in atherosclerotic plaques, or polarization in cultured BMM. Our results suggest that higher cAMP levels and the ensuing inhibition of macrophage proliferation contribute to atheroprotection in LDLR^−/−^ mice lacking p110γ. Nonetheless, p110γ deletion does not appear to be involved in apoptosis, in macrophage polarization or in T cell proliferation.

## Introduction

Atherosclerosis has traditionally been considered a disorder of cholesterol metabolism that results in lipid accumulation in the arterial wall, provoking artery wall thickening. It shares features of chronic inflammatory diseases, such as infiltration of activated immune cells into the artery wall [1], [2]. Early in the disease, oxidized low-density lipoproteins (oxLDL) that have accumulated in the intima activate endothelial cells; these secrete a number of pro-inflammatory molecules that recruit specific leukocyte types into the artery wall [3]. Monocyte/macrophages accumulate preferentially in atherosclerotic plaque, although other infiltrate components such as effector T cells, mast cells, dendritic cells and neutrophils also contribute to inflammation [3], [4], [5]. Small numbers of Foxp3^+^ regulatory T (Treg) cells, which mediate atheroprotection [5], are also present in plaques [6]. In early atherosclerotic lesions, most monocytes differentiate to macrophages due to the effect of macrophage colony-stimulating factor (M-CSF) and other mediators of innate and acquired immunity [7]. Neointimal macrophages internalize lipoproteins to become foam cells, which contribute to lipoprotein modification and retention, enhancing atherosclerosis progression [4], [7]. Macrophage and T lymphocyte activation lead to the release of additional mediators, including cytokines, chemokines and growth factors [1], [8]. This chronic inflammatory environment promotes progression of early lesions (or fatty streaks) to complex lesions (or advanced plaques) that protrude into the arterial lumen and can trigger atherothrombotic vascular disease [1], [3].

Macrophages are a heterogeneous cell population, able to adapt their physiology in response to a variety of microenvironmental situations. There are thought to be two main phenotypes; classically activated macrophages (M1) are pro-inflammatory, whereas alternatively-activated macrophages (M2) contribute to wound healing and regulation of inflammatory processes [9]. Granulocyte and macrophage colony-stimulating factor (GM-CSF)-stimulated bone marrow precursors generate cells of the M1 phenotype, whereas M-CSF promotes the M2 phenotype [10], [11]; studies describe both cell types in human and murine atherosclerotic lesions [12]. A recent report nonetheless showed predominance of infiltrating M2 macrophages in lesions in young apolipoprotein E (ApoE)-deficient mice, while M1 macrophages dominated in those of aged ApoE-deficient mice; further analysis suggested M2-to-M1 transition in the lesions [13].

Macrophage number in the lesions is controlled mainly by monocyte migration into plaques and, to a lesser extent, by macrophage apoptosis and by local macrophage proliferation [14], [15], [16]. Macrophage apoptosis has contrasting roles in plaque progression; in early lesions, it limits lesion cellularity, whereas in advanced lesions, it promotes development of the necrotic core, a high-risk factor for thrombosis [16]. Proliferation of infiltrating macrophages in early atherosclerotic plaque fosters lesion progression to a more advanced stage [14], [15], [17]. In lesions, modified LDL (low-density lipoproteins) induce GM-CSF release by infiltrating macrophages and by vascular endothelial and smooth muscle cells, which activates macrophage proliferation [17], [18], [19]. Although GM-CSF and phosphoinositide 3-kinase (PI3K) are implicated in macrophage proliferation *in vitro* [17], [20], Chang *et al*. did not detect GM-CSF by *in situ* hybridization in atherosclerotic plaque sections from ApoE-deficient mice [21]. M-CSF secreted by aortic endothelial cells also promotes macrophage proliferation in atherosclerotic lesions [14]. In murine bone marrow-derived macrophages (BMM) and in human monocytes, M-CSF induces recruitment of the PI3K p85α regulatory subunit to the M-CSF receptor, activating PI3K [22], [23], [24].

p110γ is expressed mainly in hematopoietic cells. p110γ^−/−^ mouse neutrophils have severely impaired function and migration; these mice also show reduced mast cell degranulation [25], lower thymocyte numbers and defective T cell function *in vitro* and *in vivo* [26], [27], [28]. Germ-line deletion of p110γ in ApoE^−/−^ mice attenuates murine atherosclerosis [21]. *In vitro* and *in vivo* experiments showed that p110γ is necessary for Akt activation in macrophages in response to oxLDL, atherogenic chemokines, and angiotensin II activation [21]. Pharmacological inhibition of p110γ alleviates atherosclerotic plaque development in ApoE^−/−^ and LDLR^−/−^ mice; its deletion in hematopoietic cells decreases macrophage and T cell numbers in plaque [29]. The *in vivo* mechanism underlying this reduced inflammatory cell infiltration has not been entirely clarified. Although M-CSF-stimulated p110γ-deficient BMM show reduced migration rates *in vitro* [30], p110γ deletion does not affect monocyte differentiation to macrophages [31]. p110γ regulates cyclic adenosine monophosphate (cAMP) levels in cardiomyocytes through a kinase-independent pathway that involves formation of a complex that includes p110γ, its p84/p87^PIKAP^ regulatory subunit, and the protein phosphodiesterase3B (PDE3B); this complex controls PDE3B-mediated cAMP hydrolysis [32], [33]. A similar p110γ-dependent mechanism was recently shown to mediate microglial phagocytosis via lipid kinase-independent control of cAMP [34]. It is not known whether p110γ regulates cAMP intracellular levels in macrophages. Macrophage proliferation is nonetheless affected by intracellular cAMP levels, as high levels are associated with cell cycle arrest [35]. In addition, cAMP response element binding protein (CREB) is linked to macrophage polarization to the M2 phenotype, thus connecting cAMP and M1/M2 macrophage polarization [36].

Here we examined the influence of p110γ deletion on macrophage proliferation, apoptosis and polarization in atherosclerotic plaque, and tested whether p110γ deletion contributes to lesion reduction in LDLR^−/−^ mice. We identify a p110γ function in macrophage proliferation within atherosclerotic lesions, a mechanism that contributes to atheroprotection in LDLR^−/−^ mice lacking p110γ.

## Materials and Methods

### Mice and Ethics Statement

Gene targeting in embryonic cells was used to generate LDLR^−/−^ mice [37] (Ldlr^tm1Her^ version2; Jackson Laboratories). p110γ^−/−^ mice [38] were maintained in heterozygosity. We backcrossed LDLR^−/−^ with p110γ^+/−^ and p110γ^−/−^ mice for at least 7 generations; 12- to 15-week-old mice were fed for two months with a high-fat diet (15.2% fat, containing 7.5 g/kg cholesterol (0.75% cholesterol); Ssniff Spezialdiäten GmbH, Figure S1A). Mice were bred and maintained in specific pathogen-free conditions in our animal facility; the CNB Ethics Committee for Animal Experimentation approved all animal studies (ref: 11021), in compliance with national and European Union legislation (Directive 2010/63/EU).

### Analysis of Macrophage and T cell Infiltration in Atherosclerotic Lesions

At t = 2 months of high-fat diet, mice were anesthetized (ketamine, 150 mg/kg; xylazine, 10 mg/kg; i.p.). Tail- and toe-pinch reflexes were tested to monitor adequacy of anesthesia and all efforts were made to minimize suffering. Whole blood was extracted by retro-orbital bleeding and hearts perfused with 4% paraformaldehyde. Hearts were extracted and paraffin-embedded. Some serial sections were stained by immunohistochemistry for T cells (CD3^+^), macrophages (Mac-3^+^) and regulatory T cells (Foxp3^+^) (see Supplement S1 for details).

### *In vivo* Determination of Macrophage and T cell Proliferation

Macrophage and T cell proliferation was analyzed by immunofluorescence staining of the aortic valve region in paraffin-embedded sections from LDLR^−/−^p110γ^+/−^ and LDLR^−/−^p110γ^−/−^ mice fed with a high-fat diet for two months. Markers were Mac-3 (macrophages), CD3 (T cells) and Ki67 (proliferation) (details in Supplement S1).

### *In vivo* Study of M1 and M2 Macrophages

M1 (Mac-3^+^iNOS^+^) and M2 (Mac-3^+^arginase1^+^) macrophages were analyzed by immunofluorescence staining of paraffin-embedded sections of the aortic valve region from LDLR^−/−^p110γ^+/−^ and LDLR^−/−^p110γ^−/−^ mice after a two-month high-fat diet (see Supplement S1).

### *In vivo* Determination of Lesion Apoptosis and of Vascular Smooth Muscle Cells

Lesion apoptosis was analyzed by TUNEL and cleaved caspase-3 immunofluorescence staining of the aortic valve region in paraffin-embedded sections from LDLR^−/−^p110γ^+/−^ and LDLR^−/−^p110γ^−/−^ mice fed a high-fat diet for two months. Vascular smooth muscle cell (VSMC) staining was analyzed by anti-alpha smooth muscle actin (αSMA) immunofluorescence staining of similar sections (details in Supplement S1).

### Macrophage Cell Cycle Analysis

BMM were synchronized in G0/G1 by M-CSF deprivation (36 h) and then stimulated for different times with M-CSF, collected and labeled with propidium iodide to analyze cell cycle by flow cytometry (see Supplement S1).

### Analysis of Intracellular cAMP Concentrations

BMM from LDLR^−/−^p110γ^+/−^ and LDLR^−/−^p110γ^−/−^ mice were differentiated *in vitro* and intracellular cAMP concentration determined by ELISA using the Parameter Cyclic AMP Assay kit (KGE002B, R&D Systems) (see Supplement S1).

In a second approach, BMM from LDLR^−/−^p110γ^+/−^ and LDLR^−/−^p110γ^−/−^ mice were differentiated *in vitro* and M-CSF-stimulated at several times (0, 24, 48 h). Cells were washed, lysed and protein quantified. Western blot was developed to detect protein-bound cAMP, phospho-CREB (p-CREB) and total CREB with anti-cAMP antibody (clone SPM486; Abcam, Cambridge, UK; this antibody was generated using cAMP compounds as immunogen, and a chemically linked cAMP-carrier protein for antibody screening (see Supplement S1), as well as anti-pCREB (Ser133) and -CREB (both from Cell Signaling, Danvers, MA). β-actin was used as loading control (clone AC-15, Sigma); band intensity was quantified using ImageJ software. As a positive control, BMM from p110γ^+/−^ mice were differentiated *in vitro* and forskolin (FSK)-stimulated, and cAMP was detected in Western blot (see Supplement S1).

### M1 and M2 Macrophage Differentiation

After BMM differentiation (see Supplement S1), cells were plated in 6-well plates (1–2 × 10^6^ cells/well) and incubated (24 h) in complete DMEM (10% FBS, antibiotics, 2 mM glutamine) and 10 ng/ml IL-4 (Peprotech, Rocky Hill, NJ) for M2 macrophage differentiation, or 10 ng/ml IFNγ (Peprotech) and 100 ng/ml lipopolysaccharide (LPS; Sigma) for M1 macrophage differentiation. Cells were washed in cold PBS, resuspended in 0.6 to 1 ml TRI Reagent (Sigma), and stored at –80°C for RNA extraction.

### qRT-PCR Analysis of M1 and M2 Macrophage Marker Expression

RNA was extracted from M1- or M2-differentiated BMM from p110γ^+/−^ and p110γ^−/−^ mice. qRT-PCR was performed using specific primers for M1 (iNOS, IL-12) and M2 markers (arginase1, YM1, IL-10) (see Supplement S1).

### Statistical Analysis

Data are represented as mean ± SD. Most statistical analyses were performed using Student’s *t*-test to compare distinct parameters in two independent mouse groups (LDLR^−/−^p110γ^+/−^ and LDLR^−/−^p110γ^−/−^ or p110γ^+/−^ and p110γ^−/−^). Where indicated, data obtained by counting and small sample analysis were compared by the Poisson test. In all cases, differences were considered significant for p<0.05 (*p<0.05, **p<0.01).

## Results

### Lack of p110γ in LDLR^−/−^ Mice Reduces Atherosclerosis Burden

**Figure 1:**
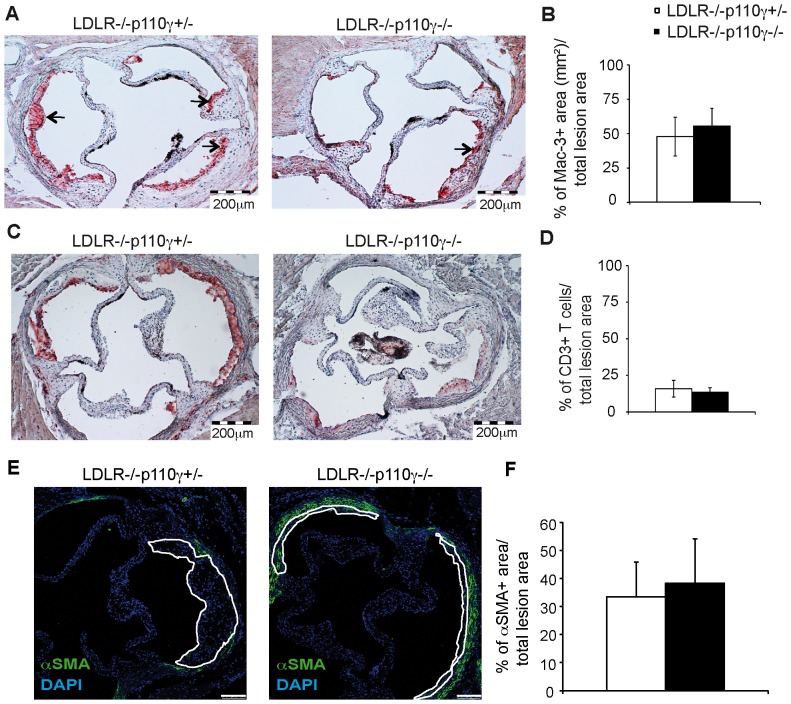
Macrophage and T cell infiltration in lesions of LDLR^−/−^p110γ^−/−^ compared to LDLR^−/−^p110γ^+/−^ mice. Aortic sinus sections were studied in LDLR^−/−^p110γ^+/−^ (females, *n* = 6) and LDLR^−/−^p110γ^−/−^ mice (females, *n* = 7) after two months on a high-fat diet. (**A**) Representative photomicrographs of Mac-3^+^ cells in aortic sinus sections after immunohistochemical staining. Bar = 200 μm. Arrows indicate Mac-3^+^ area. (**B**) Percentage of Mac-3^+^-stained area relative to total lesion area, quantified with ImageJ software. (**C**) Representative photomicrographs of CD3^+^ cells in aortic sinus sections after immunohistochemical staining. Bar = 200 μm. (**D**) Percentage of CD3^+^ cells relative to total lesion area, quantified with ImageJ. (**E**) Representative photomicrographs of immunofluorescent staining for vascular smooth muscle cells (αSMA^+^) in aortic sinus sections from LDLR^−/−^p110γ^+/−^ and LDLR^−/−^p110γ^−/−^ mice after two months on a high-fat diet (*n* = 6 females/genotype). Bar = 100 μm. (**F**) Percentage of αSMA^+^ area relative to total lesion area, quantified with ImageJ. Mean ± SD. Student’s *t*-test.

To determine the effect of PI3K p110γ deletion on apoptosis, macrophage proliferation and polarization in atherosclerotic lesions, we generated LDLR^−/−^ mice lacking one (LDLR^−/−^p110γ^+/−^) or both p110γ alleles (LDLR^−/−^p110γ^−/−^). Mice of both genotypes were fed a high-fat diet for two months to induce the disease. We analyzed atherosclerosis burden by planimetric analysis of hematoxylin/eosin-stained cross-sections of the aortic sinus. Lesion size was smaller in LDLR^−/−^p110γ^−/−^ than in LDLR^−/−^p110γ^+/−^ mice (Supplement S1, Figure S1B, S1C). To evaluate the number of infiltrating macrophages, T cells and regulatory T cells in p110γ deletion conditions, we stained aortic sinus sections from these mice with macrophage-, T cell- and Treg-specific markers (Mac-3^+^, CD3^+^ and Foxp3^+^, respectively). We observed a significant decrease in Mac-3^+^-stained area in LDLR^−/−^p110γ^−/−^ compared to LDLR^−/−^p110γ^+/−^ lesions (Figure 1A, S1D). The absolute number of CD3^+^ T cells was similarly reduced in LDLR^−/−^p110γ^−/−^ compared to LDLR^−/−^p110γ^+/−^ lesions (Figure 1C, S1E). Foxp3^+^ staining was negligible in LDLR^−/−^p110γ^+/−^ lesions (0–3 cells/section) and absent in LDLR^−/−^p110γ^−/−^ lesions (*n* = 8 mice/genotype, Table S1). Nonetheless, because lesions were smaller in LDLR^−/−^p110γ^−/−^ than in LDLR^−/−^p110γ^+/−^ mice, Mac-3^+^-stained area and CD3^+^ cell number relative to total lesion area were similar in LDLR^−/−^p110γ^−/−^ and LDLR^−/−^p110γ^+/−^ mice (Figure 1B, 1D). We also stained vascular smooth muscle cell (VSMC) using αSMA antibody (Figure 1E). As indicated by the ratio of αSMA^+^ area/total lesion area, we found no differences between LDLR^−/−^p110γ^−/−^ and LDLR^−/−^p110γ^+/−^ mice (Figure 1F).

### p110γ Deficiency Reduces Macrophage but not T cell Proliferation in Atherosclerotic Lesions

**Figure 2:**
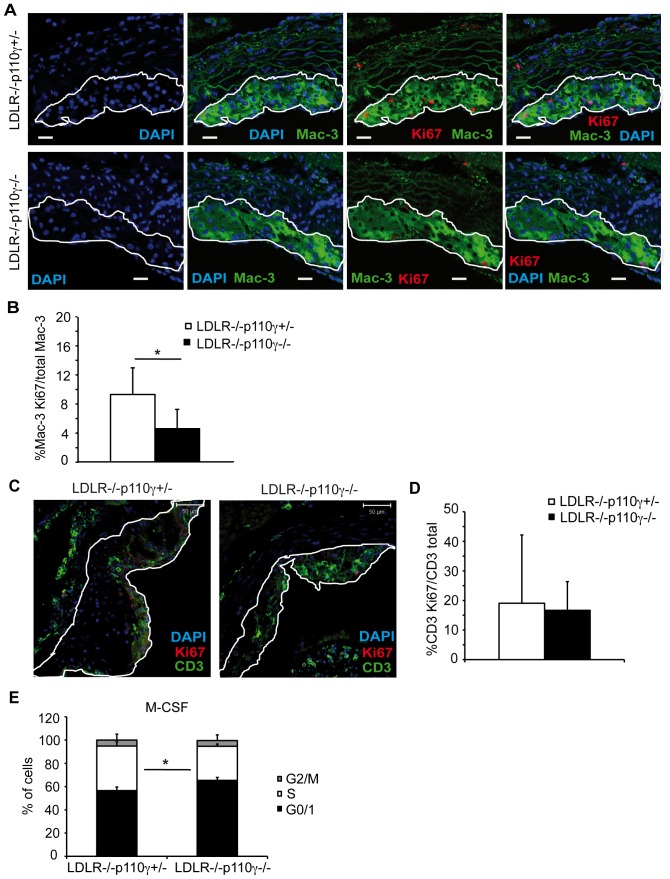
Macrophage proliferation in aortic plaque is impaired in LDLR^−/−^p110γ^−/−^ mice whereas T cell proliferation is unaffected. Atherosclerotic plaques were studied in LDLR^−/−^p110γ^+/−^ (female, *n* = 5) and LDLR^−/−^p110γ^−/−^ (female, *n* = 6) mice after two months on a high-fat diet. (**A**) Representative photomicrographs of immunofluorescent staining for macrophage proliferation in aortic sections. Bar = 30 μm. (**B**) Percentage of proliferating relative to total macrophages in lesion area. (**C**) Representative photomicrographs of immunofluorescent staining for T cell proliferation in aortic sections. Bar = 50 μm. (**D**) Percentage of proliferating T cells relative to total T cells in lesion area, quantified with ImageJ. (**E**) Percentage of bone marrow-derived macrophages (BMM) in G2/M, S and G0/G1 phases at 26 h post-M-CSF stimulation in LDLR^−/−^p110γ^+/−^ and LDLR^−/−^p110γ^−/−^ mice (*n* = 3 experiments, each with a pool of 3 mice/genotype). Mean ± SD; Student’s *t*-test, p<0.05 (for B, D, E).

Macrophage proliferation in lesions enhances atherosclerosis progression to more advanced disease stages [15]. To determine whether the reduced atherosclerosis burden in LDLR^−/−^p110γ^−/−^ mice correlated with cell proliferation defects in lesions, we performed double immunofluorescence experiments in aortic cross-sections from high-fat diet-fed mice to test whether p110γ deficiency affected macrophage and T cell *in situ* proliferation (as assessed by Ki67 expression). These studies showed a significant reduction in the number of proliferating neointimal macrophages in LDLR^−/−^p110γ^−/−^ compared to LDLR^−/−^p110γ^ +/−^ mice (Figure 2A, 2B). In contrast, p110γ deletion did not affect T cell proliferation (Figure 2C, 2D).

M-CSF is thought to play an important role in inducing macrophage proliferation in atherosclerotic lesions [14]. Cell cycle analysis of *in vitro-*differentiated BMM from LDLR^−/−^p110γ^+/−^ and LDLR^−/−^p110γ^−/−^ mice allowed us to identify the proportion of cells in G0/G1, S and G2/M phases at various times post-stimulation with M-CSF. The proportion of S phase cells was reduced in LDLR^−/−^p110γ^−/−^ compared to LDLR^−/−^p110γ^+/−^ macrophages at 26 h after M-CSF-stimulation (Figure 2E), suggesting a role for p110γ in macrophage cell cycle progression. In contrast, cell cycle assays to study *in vitro* BMM proliferation in response to GM-CSF showed no differences between p110γ^+/−^ and p110γ^−/−^ BMM (Figure S2).

### Lesion Apoptosis is Unaffected by p110γ Deletion

**Figure 3:**
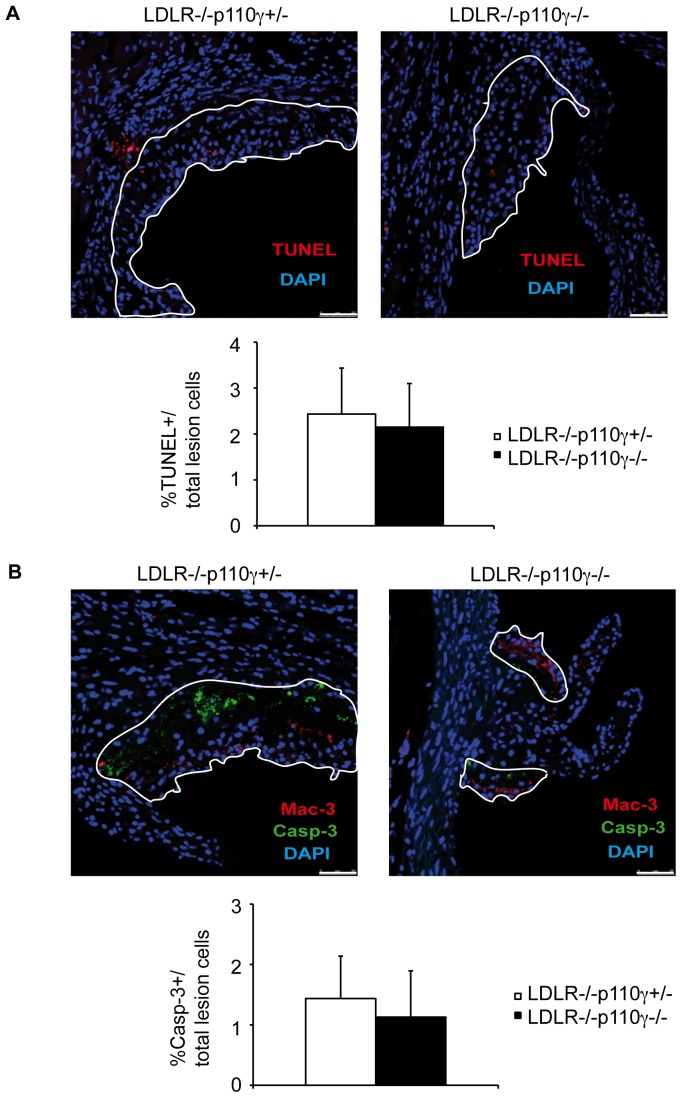
Lesion apoptosis is unaffected by p110γ deletion. Atherosclerotic plaques were analyzed in LDLR^−/−^p110γ^+/−^ and LDLR^−/−^p110γ^−/−^ mice after a two-month high-fat diet. (**A**) Representative photomicrographs of TUNEL immunofluorescent staining for lesion apoptosis in aortic sections from LDLR^−/−^p110γ^+/−^ (*n* = 8) and LDLR^−/−^p110γ^−/−^ mice (*n* = 8) (top); percentage of TUNEL^+^ relative to total cells in the delimited lesion area (bottom). Bar = 50 μm. (**B**) Representative photomicrographs of cleaved caspase-3 immunofluorescent staining for lesion apoptosis in aortic sections from LDLR^−/−^p110γ^+/−^ (*n* = 5) and LDLR^−/−^p110γ^−/−^ mice (*n* = 5) (top); percentage of cleaved caspase-3^+^ relative to total cells in the delimited lesion area (bottom). Bar = 50 μm. Mean ± SD; Student’s *t*-test.

Macrophage apoptosis has been implicated in plaque progression [16]. We measured total apoptosis in lesions by TUNEL (Figure 3A) and cleaved caspase-3 (Figure 3B) immunofluorescent staining of aortic sinus sections from LDLR^−/−^p110γ^+/−^ and LDLR^−/−^p110γ^−/−^ mice. Lesion area was delimited for TUNEL staining with the help of smooth muscle cells (SMC), which limit lesion area and are autofluorescent, and for cleaved caspase-3 staining by adding Mac-3 staining to the SMC guide; some lesion apoptotic cells are not Mac-3^+^. We detected a tendency toward lower apoptotic rates in LDLR^−/−^p110γ^−/−^ compared to LDLR^−/−^p110γ^+/−^ mice (Figure 3A, 3B), although the differences were not significant.

### Reduced LDLR^−/−^p110γ^−/−^ Macrophage Proliferation Correlates with Increased Intracellular Basal cAMP Levels

**Figure 4:**
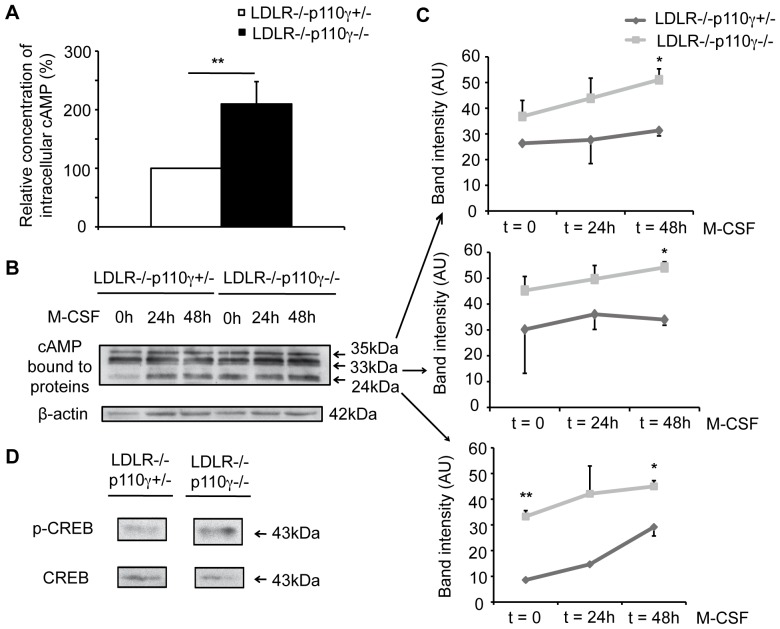
Intracellular cAMP levels are higher in LDLR^-/−^p110γ^−/−^ than in LDLR^−/−^p110γ^+/−^macrophages. LDLR^−/−^p110γ^+/−^ and LDLR^−/−^p110γ^−/−^ BMM were allowed to differentiate and were lysed. (**A**) ELISA was used to determine intracellular cAMP levels in unstimulated BMM lysates (t = 0). The graph shows basal intracellular cAMP levels in LDLR^−/−^p110γ^−/−^ vs. LDLR^−/−^p110γ^+/−^ BMM (*n* = 4 experiments). Mean ± SD; Student’s *t*-test, p<0.01. (**B**) Western blot of BMM extracts to detect protein-bound cAMP after M-CSF stimulation (0, 24 and 48 h). (**C**) Bands in (B) were quantified with ImageJ and measured as arbitrary units (AU). Mean ± SD of four independent experiments, each with one mouse/genotype. Student’s *t*-test, p<0.05 and p<0.01. (**D**) Western blot of BMM extracts to detect p-CREB and total CREB (t = 0).

Since p110γ regulates cAMP levels in cardiomyocytes and microglia [32], [33], [34] we tested whether this is the case in macrophages, using ELISA to measure intracellular cAMP levels in LDLR^−/−^p110γ^+/−^ and LDLR^−/−^p110γ^−/−^ mouse BMM. Basal cAMP levels were higher in LDLR^−/−^p110γ^−/−^ compared to LDLR^−/−^p110γ^+/−^ BMM (Figure 4A). In an alternative approach, we measured differences in protein-bound cAMP in Western blot, using an antibody that recognizes protein-bound cAMP [39]. In macrophages, this antibody recognizes major three bands of 24, 33 and 35 kDa, as determined when BMM from p110γ^+/−^mice were differentiated *in vitro* and FSK-stimulated, and cAMP detected by Western blot (Figure S3). Again, basal cAMP levels were significantly higher in BMM lysates from LDLR^−/−^p110γ^−/−^ than from LDLR^−/−^p110γ^+/−^ mice and this difference was sustained after M-CSF stimulation (Figure 4B, 4C).

In macrophages, signals that increase intracellular cAMP induce phosphorylation of cAMP response element-binding protein (CREB) [40]. As an alternative measurement of cAMP levels, we tested CREB phosphorylation status in LDLR^−/−^p110γ^+/−^ and LDLR^−/−^p110γ^−/−^ mouse BMM. Coincident with the increased cAMP detected in the protein lysates, we found higher basal p-CREB levels in LDLR^−/−^p110γ^−/−^ mouse BMM (Figure 4D). The data suggest that lack of p110γ in macrophages promotes intracellular cAMP accumulation, which correlates with G0/G1 cell cycle arrest in LDLR^−/−^p110γ^−/−^ mouse BMM (Figure 2E) since high cAMP levels are associated with cell cycle arrest [35].

### p110γ Deficiency does not Affect Macrophage Polarization to M1 and M2 Phenotypes

**Figure 5:**
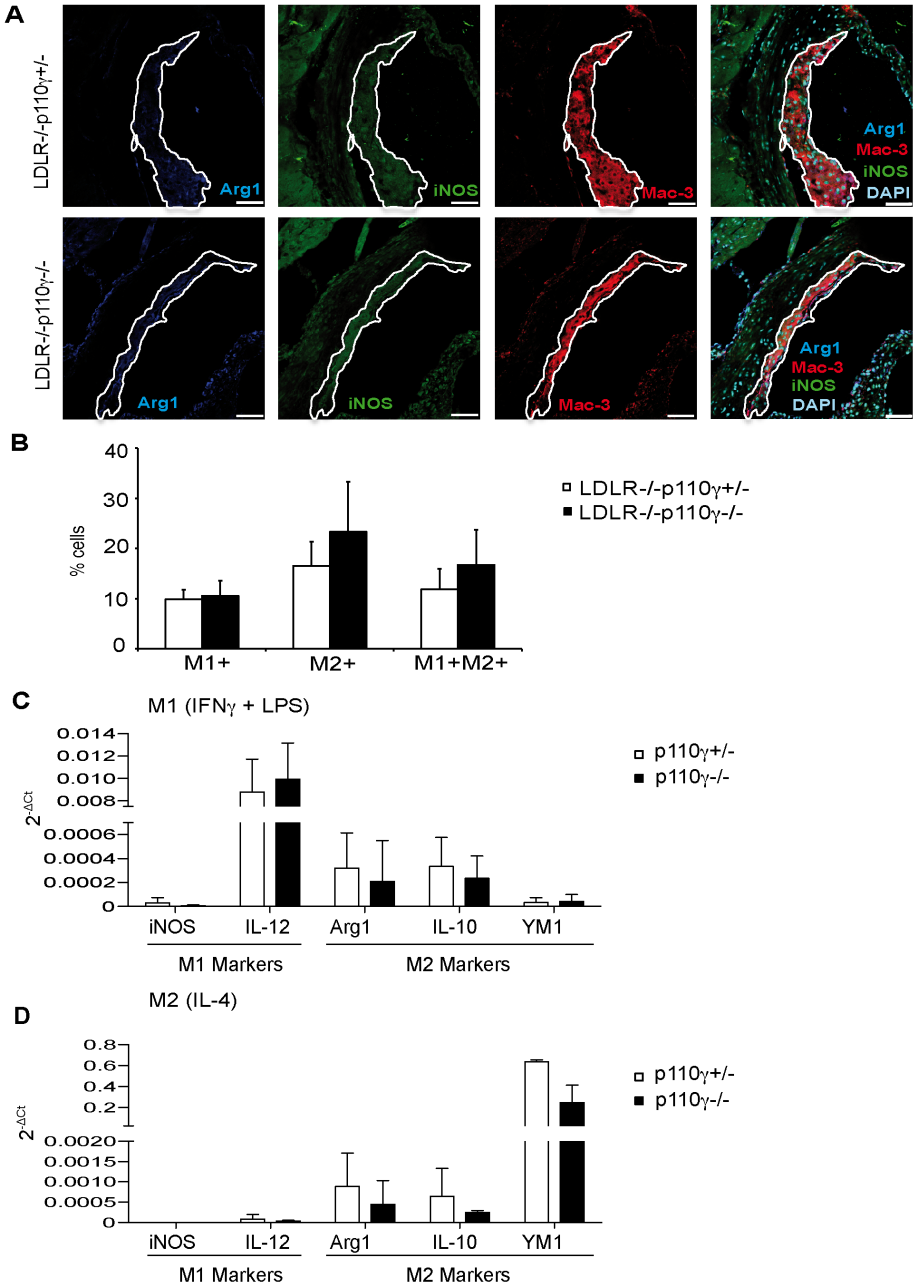
M1 and M2 macrophage populations are similar in LDLR^−/−^p110γ^+/−^ and LDLR^-/−^p110γ^−/−^ mice. (**A**) Representative photomicrographs of immunofluorescent-stained M1 (iNOS^+^) and M2 (arginase1^+^) macrophages in aortic sinus sections from LDLR^−/−^p110γ^+/−^ and LDLR^−/−^p110γ^−/−^ mice fed a two-month high-fat diet (*n* = 5/genotype). Bar = 50 μm. (**B**) Quantification of the percentage of M1^+^ (iNOS^+^), M2^+^ (arginase1^+^) and M1^+^M2^+^ (iNOS^+^arginase1^+^) macrophage subsets relative to total macrophages in aortic plaques. Mean ± SD; Student’s *t*-test. Expression of M1 (iNOS, IL-12) and M2 markers (arginase1, YM1, IL-10) was analyzed by qRT-PCR in BMM stimulated with (**C**) IFNγ+LPS (M1) *(n* = 4 experiments, 3 mice/genotype) or (**D**) IL-4 (M2) *(n* = 4 experiments, 3 mice/genotype); marker expression is shown as RQ values. RQ = 2^–ΔCt^.

cAMP is linked to macrophage transition to the M2 phenotype [36]. As we observed increased cAMP levels in LDLR^−/−^p110γ^−/−^ BMM, we analyzed macrophage polarization in atherosclerotic lesions. Immunofluorescence experiments showed no significant differences in the percentage of M1 (iNOS^+^) and M2 (arginase1^+^) macrophages in aortic sinus cross-sections from LDLR^−/−^p110γ^+/−^ and LDLR^-/−^p110γ^−/−^ mice (Figure 5A, 5B), although there was a tendency toward more M2 macrophages in LDLR^−/−^p110γ^−/−^ mice. Consistent with this finding, qRT-PCR studies of p110γ^+/−^ and p110γ^−/−^ mouse BMM stimulated *in vitro* (24 h) towards the M1 (IFNγ+LPS) or M2 phenotypes (IL-4) showed no significant differences in M1 (iNOS, IL-12) and M2 (arginase1, IL-10, YM1) marker expression (Figure 5C, 5D).

## Discussion

PI3K p110γ is implicated in atherosclerosis, as its genetic deletion in ApoE^−/−^ mice leads to reduced plaque size and impaired activation of the PI3K/Akt pathway in neointimal macrophages [21]. Pharmacological inhibition of p110γ reduces atherosclerosis in ApoE^−/−^ and LDLR^−/−^ mice, and reconstitution of LDLR^−/−^ mice with p110γ^−/−^ mouse bone marrow leads to decreased T cell and monocyte infiltration in atherosclerotic plaques [29]. Whether p110γ deletion also contributes to local macrophage proliferation and apoptosis nonetheless remains unclear, as does the role of p110γ in M1/M2 macrophage differentiation. In this study, we approached these questions by analyzing atherosclerosis development in LDLR^−/−^p110γ^−/−^ mice.

Immune cell infiltration is an important step in plaque formation and progression [5]. In agreement with previous studies [21], [29], we found smaller aortic sinus lesions in LDLR^−/−^p110γ^−/−^ than in LDLR^−/−^p110γ^+/−^ mice; our experiments showed reduced Mac-3^+^-stained lesion area and absolute numbers of CD3^+^ T cells in LDLR^−/−^p110γ^−/−^ vs. LDLR^−/−^p110γ^+/−^ mice. As atherosclerotic lesions were smaller in LDLR^−/−^p110γ^−/−^ than in LDLR^−/−^p110γ^+/−^ mice, however, macrophage area and T cell number relative to total lesion area were similar in the two strains. These data coincide with the unchanged macrophage density in ApoE^−/−^p110γ^−/−^ compared to ApoE^−/−^p110γ^+/−^ lesions [21], but differ from the data for irradiated LDLR^−/−^ mice reconstituted with p110γ^−/−^ bone marrow, which showed a marked decrease in macrophage and T cell infiltration (40% reduction) relative to total lesion area [29]. The differences between the data from LDLR^−/−^p110γ^−/−^ and ApoE^−/−^p110γ^−/−^ mice, and those from irradiated LDLR^−/−^ mice reconstituted with p110γ^−/−^ bone marrow could reflect a p110γ function in lymphoid organ reconstitution, in addition to its role in infiltration. Flow cytometry analysis showed similar percentages of circulating immune cell populations (before and after high-fat diet) in LDLR^−/−^p110γ^+/−^ and LDLR^−/−^p110γ^−/−^ mice, except for neutrophils, which were increased in LDLR^−/−^p110γ^−/−^ mice (Supplement S1, Figure S4), coinciding with the p110γ^−/−^ mouse phenotype [38].

Low Treg cell numbers in atherosclerotic lesions are reported in LDLR^−/−^ mice [41] and humans [6]. We observed few Foxp3^+^ cells in early lesions of LDLR^−/−^p110γ^+/−^ and LDLR^−/−^p110γ^−/−^ mice (Table S1), which did not permit differentiation between genotypes. Because oxLDL downregulates Foxp3 expression in mouse effector cells *in vitro* [42], oxLDL accumulation in lesions could explain the low Foxp3^+^ cell numbers. oxLDL in arterial walls might inhibit Treg cell function, contributing to chronicity [42]. Our data indicate that p110γ does not affect Treg cell infiltration into atherosclerotic plaques.

Macrophage proliferation in lesions promotes more rapid atherosclerosis progression [15]. Our studies of aortic sections showed a lower percentage of proliferating macrophages in LDLR^−/−^p110γ^−/−^ than in LDLR^−/−^p110γ^+/−^ mice, although there were no differences in T cell proliferation between the two genotypes. We complemented *in vivo* analysis of macrophage proliferation with *in vitro* experiments using BMM. Whereas GM-CSF-induced proliferation was similar in BMM from p110γ^+/−^ and p110γ^−/−^ mice (Figure S2), proliferation was reduced and S phase entry delayed in M-CSF-stimulated LDLR^−/−^p110γ^-/−^ compared with control LDLR^−/−^p110γ^+/−^ BMM (Figure 2E), reflecting a specific p110γ function in these processes after M-CSF signaling.

A role for p110γ is proposed for proliferation of T cells [26], [43], B cells [44], cancer cells from medulloblastoma [45] and hepatocellular carcinoma [46], although the underlying mechanisms remain largely unknown. In macrophages, M-CSF-mediated proliferation is inhibited when intracellular cAMP levels increase [47], leading to cell cycle arrest in G1 [35], [48]. Because p84/p87^PIKAP^, the regulatory subunit of class I_B_ PI3K, is expressed at high levels in mouse macrophages, and the macromolecular complex p84/p110γ/PDE3B reduces cAMP levels [32], [33], p110γ might regulate basal cAMP levels in macrophages through formation of this complex. In microglial cells, p110γ controls basal intracellular cAMP and p-CREB levels [34]. M-CSF-stimulated LDLR^−/−^p110γ^−/−^ BMM proliferated less than control LDLR^−/−^p110γ^+/−^ BMM (Figure 2E); we thus propose that in the case of LDLR^−/−^p110γ^+/−^ macrophages, formation of a complete p84/p110γ/PDE3B complex maintains low basal intracellular cAMP levels, allowing M-CSF-induced proliferation. In LDLR^−/−^p110γ^-/−^ macrophages, formation of an incomplete p84/p110γ/PDE3B complex, which normally represses cAMP production, leads to higher basal cAMP levels, which reduce M-CSF-induced proliferation. The higher basal cAMP levels in LDLR^−/−^p110γ^−/−^ than in LDLR^−/−^p110γ^+/−^ BMM (Figure 4) correlated with their proliferation rates, which supports this view.

Apoptosis was measured by TUNEL (Figure 3A) and cleaved caspase-3 (Figure 3B) immunofluorescent staining. Apoptosis was unaffected in LDLR^-/−^p110γ^+/−^ compared to LDLR^−/−^p110γ^−/−^ mouse lesions (Figure 3), although there was a tendency toward less apoptosis in LDLR^-/−^p110γ^−/−^ lesions. This tendency might reflect a delay in lesion progression in LDLR^-/−^p110γ^−/−^ mice, which have less advanced plaques than LDLR^−/−^p110γ^+/−^ mice after the same time on a high fat diet. Apoptosis rates after the two-month diet were low in LDLR^−/−^p110γ^+/−^ and LDLR^−/−^p110γ^−/−^ mice, possibly because lesions developed at this stage are still early lesions and apoptotic cells would be correctly efferocytosed, a process that worsens with lesion severity and leads to apoptotic cell accumulation [49]. We stained αSMA to detect VSMC in LDLR^-/−^p110γ^+/−^ and LDLR^-/−^p110γ^−/−^ aortic sections, and found no differences in the percentage of αSMA^+^ staining relative to total lesion area in either genotype (Figure 1E, 1F). As VSMC are not reduced in LDLR^-/−^p110γ^−/−^ plaques, these cells might not have the proliferative defect found in LDLR^-/−^p110γ^−/−^ macrophages; this suggests that the proliferative disorder is cell type-specific.

Macrophages undergo classical activation in response to LPS and IFNγ, as part of the Th1 response (M1), or alternative activation in response to IL-4 as part of the Th2 response (M2) [50]. Advanced lesions in old ApoE-null mice show a prevalence of M1 over M2 macrophages, suggesting that the M2 phenotype is atheroprotective [13]. In activated primary macrophages, expression of M2-related genes (*Arg-1*, *Il-10*, *Il13ra*, *Msr1*) depends on CREB-induced expression of *Cebpb* (a gene that encodes a protein important for macrophage antibacterial activity) [36]. High cAMP levels could thus be linked to M2 macrophage polarization. Our data from LDLR^−/−^p110γ^+/−^ and LDLR^−/−^p110γ^−/−^ mice showed no significant differences in the relative number of M1 and M2 macrophages in atherosclerotic lesions, although there was a tendency toward increased percentages of M2 macrophages in LDLR^−/−^p110γ^−/−^ compared to LDLR^−/−^p110γ^+/−^ mice (Figure 5B). Likewise, *in vitro* macrophage polarization was unaffected when we compared p110γ^+/−^ and p110γ^−/−^ BMM, which showed similar M1 and M2 marker expression.

Our results suggest that in addition to affecting macrophage infiltration [21], [29], p110γ deletion specifically alters *in situ* macrophage proliferation in atherosclerotic lesions. In p110γ^−/−^ macrophages, higher basal cAMP levels reduce M-CSF-induced proliferation. In contrast, p110γ has no role in M1/M2 macrophage differentiation or in apoptosis. Our findings confirm a mechanism by which atherosclerotic lesions can be reduced, and highlight p110γ as a potential target for treatment of inflammatory diseases.

## Supporting Information

Figure S1 **Mice lacking LDLR and PI3K p110γ show smaller atherosclerotic lesions than controls.** Lesion progression was studied in LDLR^−/−^p110γ^+/−^ and LDLR^−/−^p110γ^−/−^ mice before (t = 0) and after (t = 2 months) high-fat diet treatment. (**A**) Total serum cholesterol, HDL- and LDL-cholesterol and triglycerides were measured. t = 0, *n* = 10 mice/genotype; t = 2 months, *n* = 6 mice/genotype. Mean ± SD. Student’s *t*-test, p<0.05 (**B**) Representative photomicrographs of hematoxylin/eosin-stained aortic sinus sections from LDLR^−/−^p110γ^+/−^ and LDLR^−/−^p110γ^−/−^ female mice. Lesion area is delimited. Bar = 200 μm. (**C**) Quantitative analysis of lesion size in the aortic sinus of LDLR^−/−^p110γ^+/−^ (*n* = 6) and LDLR^−/−^p110γ^−/−^ mice (*n* = 6) using ImageJ. Mean ± SD. Student’s *t*-test, p<0.05. (**D**) Mac-3^+^ area per aortic sinus section, quantitated with ImageJ. Mean ± SD; Student’s *t*-test, p<0.05. (**E**) Absolute numbers of lesion CD3^+^ cells per aortic sinus section, quantitated with ImageJ. Mean ± SD; Poisson test, p<0.01.
(TIF)

Figure S2 **Proliferation of** **GM-CSF-stimulated macrophages is unaffected by p110γ deficiency.** Percentage of BMM in cell cycle S phase at various times post-GM-CSF stimulation in p110γ^+/−^ and p110γ^−/−^ BMM (*n* = 2 experiments, each with a pool of 3 mice/genotype).
(TIF)

Figure S3 **Intracellular cAMP levels in p110γ^+/−^ BMM increase after forskolin stimulation.** Western blot of BMM extracts from p110γ^+/−^ mice, to detect protein-bound cAMP after forskolin (FSK) stimulation (0, 24 and 48 h).
(TIF)

Figure S4 **Immune cell populations and M-CSF concentration in peripheral blood from LDLR^−/−^p110γ^+/−^ and LDLR^−/−^p110γ^−/−^ mice.** Peripheral blood was extracted from LDLR^−/−^p110γ^+/−^ and LDLR^−/−^p110γ ^−/−^ mice before (t = 0) and after (t = 2 months) on a high-fat diet. Flow cytometry staining was used to detect T cells (CD3^+^) **(A)**, inflammatory monocytes (Ly6C^hi^) **(B)** and granulocytes (Gr1^+^) **(C)** (*n* = 7 mice/genotype, t = 0; *n* = 4 mice/genotype, t = 2 months). Mean ± SD, Student’s *t*-test, p<0.05 and p<0.01. **(D)** In serum from peripheral blood obtained as above, M-CSF levels were determined by ELISA using the Milliplex Kit (Millipore). Mean ± SD. Student’s *t*-test.
(TIF)

Table S1 **Foxp3^+^ regulatory T cells infiltrate in atherosclerotic lesions from LDLR^−/−^p110γ^+/−^ and LDLR^-/−^p110γ^−/−^ mice.** Quantitative analysis of Foxp3^+^ cells per aortic sinus section of indicated mice (*n* = 8/genotype). Results show mean ± SD.
(DOC)

Supplement S1 **Supporting Materials and Methods, Results and References.**
(DOC)
