# Supplementary figures and images for: PI3K p110γ Deletion Attenuates Murine Atherosclerosis by Reducing Macrophage Proliferation but Not Polarization or Apoptosis in Lesions
Source: PLoS One. 2013 Aug 22;8(8):e72674. doi: 10.1371/journal.pone.0072674 (PMC3750002; doi:10.1371/journal.pone.0072674)

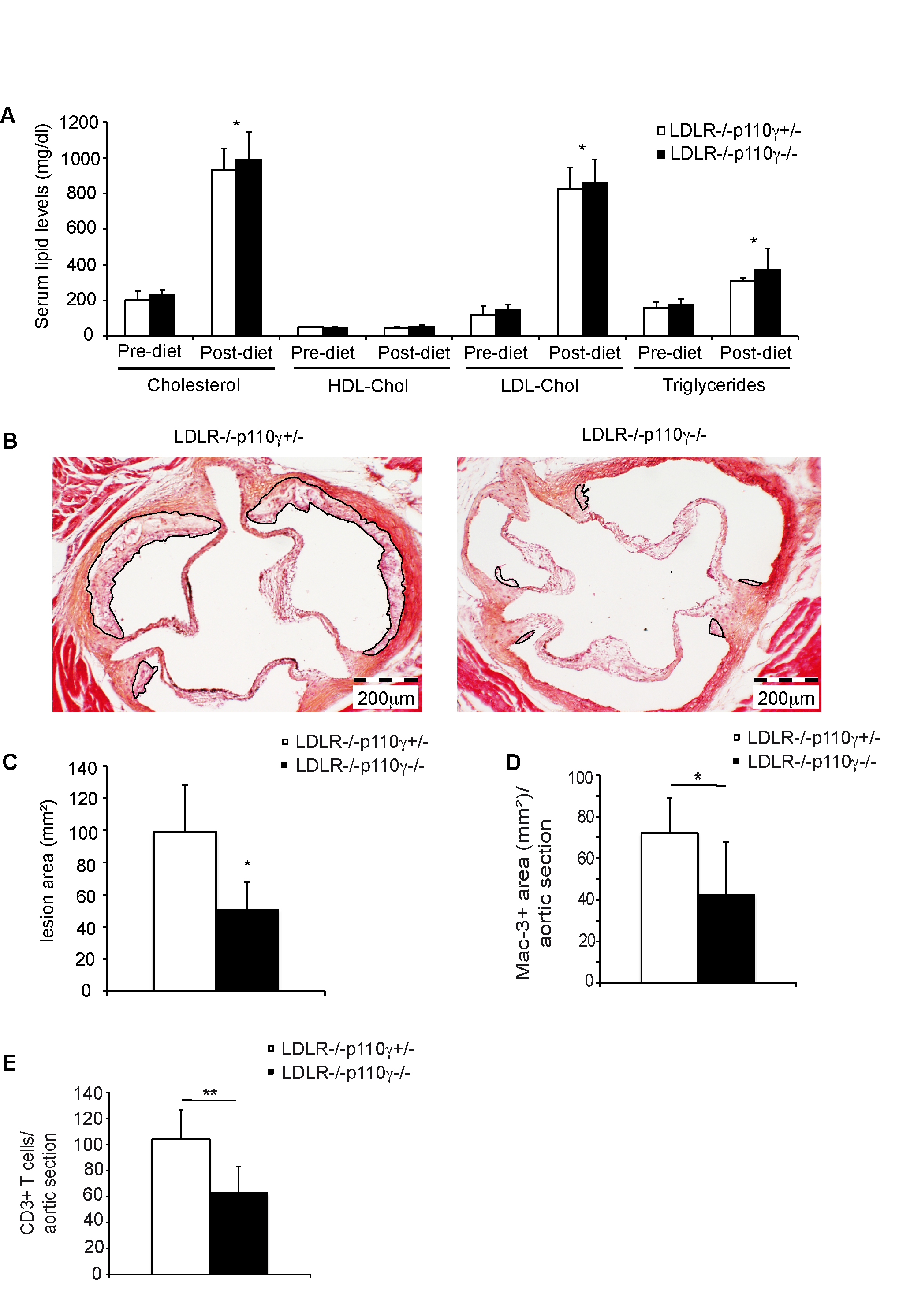

Supplement: Figure S1 — Mice lacking LDLR and PI3K p110γ show smaller atherosclerotic lesions than controls. Lesion progression was studied in LDLR−/−p110γ+/− and LDLR−/−p110γ−/− mice before (t = 0) and after (t = 2 months) high-fat diet treatment. (A) Total serum cholesterol, HDL- and LDL-cholesterol and triglycerides were measured. t = 0, n = 10 mice/genotype; t = 2 months, n = 6 mice/genotype. Mean ± SD. Student’s t-test, p<0.05 (B) Representative photomicrographs of hematoxylin/eosin-stained aortic sinus sections from LDLR−/−p110γ+/− and LDLR−/−p110γ−/− female mice. Lesion area is delimited. Bar = 200 μm. (C) Quantitative analysis of lesion size in the aortic sinus of LDLR−/−p110γ+/− (n = 6) and LDLR−/−p110γ−/− mice (n = 6) using ImageJ. Mean ± SD. Student’s t-test, p<0.05. (D) Mac-3+ area per aortic sinus section, quantitated with ImageJ. Mean ± SD; Student’s t-test, p<0.05. (E) Absolute numbers of lesion CD3+ cells per aortic sinus section, quantitated with ImageJ. Mean ± SD; Poisson test, p<0.01. (TIF) [file pone.0072674.s001.tif]

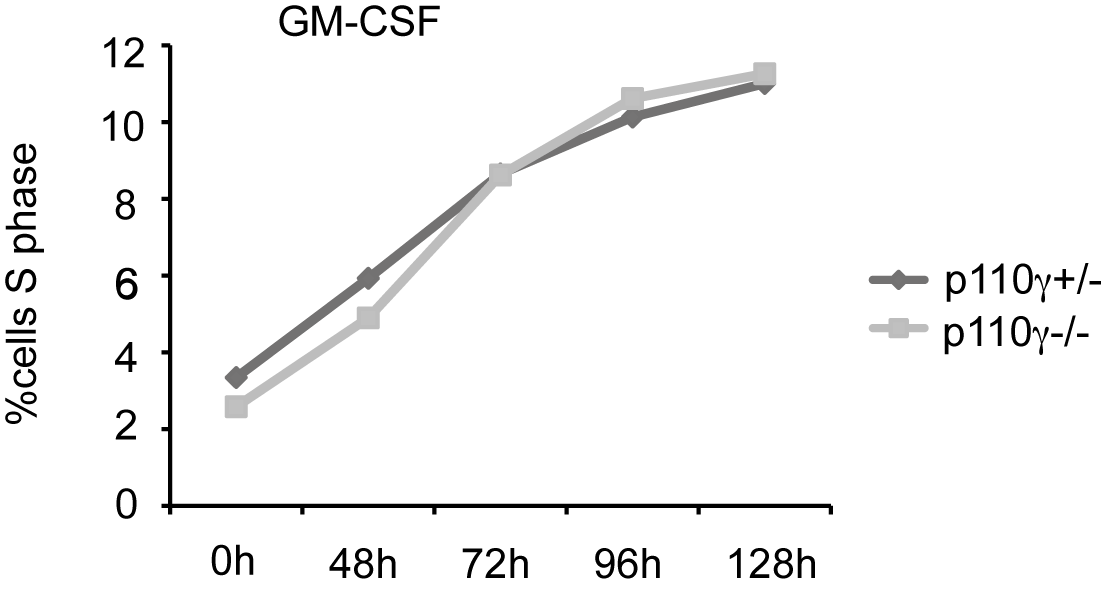

Supplement: Figure S2 — Proliferation of GM-CSF-stimulated macrophages is unaffected by p110γ deficiency. Percentage of BMM in cell cycle S phase at various times post-GM-CSF stimulation in p110γ+/− and p110γ−/− BMM (n = 2 experiments, each with a pool of 3 mice/genotype). (TIF) [file pone.0072674.s002.tif]

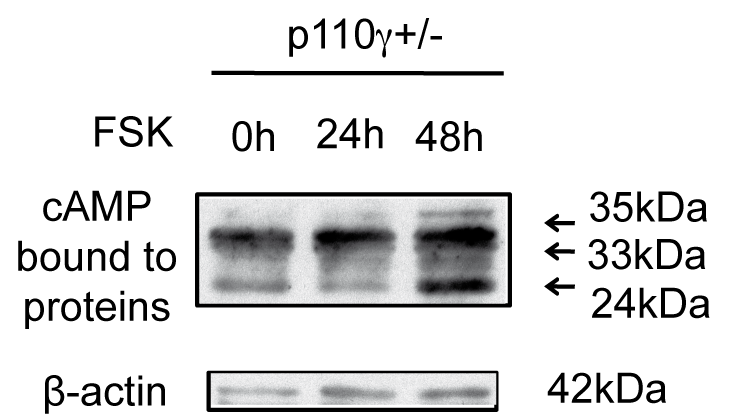

Supplement: Figure S3 — Intracellular cAMP levels in p110γ+/− BMM increase after forskolin stimulation. Western blot of BMM extracts from p110γ+/− mice, to detect protein-bound cAMP after forskolin (FSK) stimulation (0, 24 and 48 h). (TIF) [file pone.0072674.s003.tif]

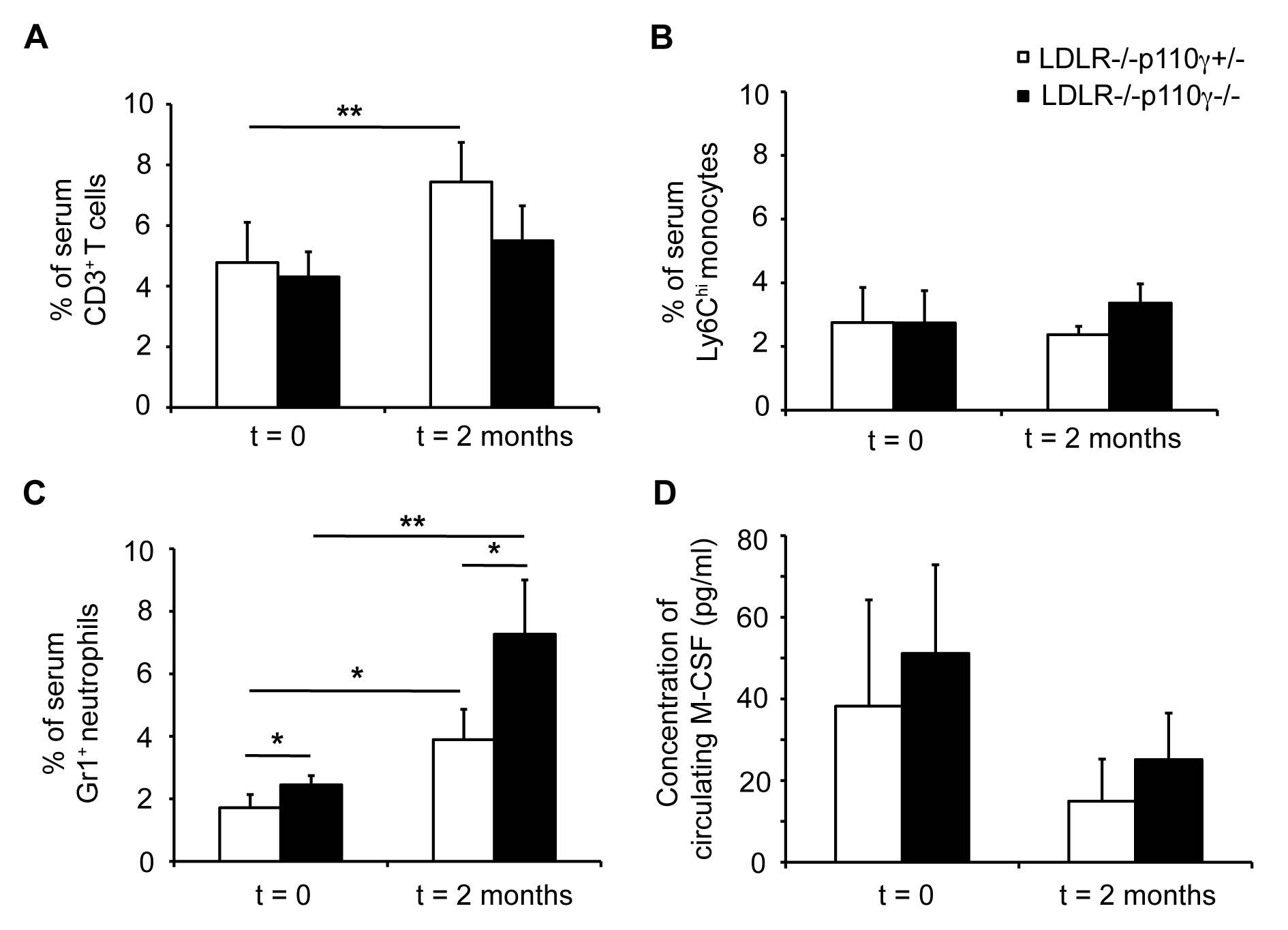

Supplement: Figure S4 — Immune cell populations and M-CSF concentration in peripheral blood from LDLR−/−p110γ+/− and LDLR−/−p110γ−/− mice. Peripheral blood was extracted from LDLR−/−p110γ+/− and LDLR−/−p110γ −/− mice before (t = 0) and after (t = 2 months) on a high-fat diet. Flow cytometry staining was used to detect T cells (CD3+) (A), inflammatory monocytes (Ly6Chi) (B) and granulocytes (Gr1+) (C) (n = 7 mice/genotype, t = 0; n = 4 mice/genotype, t = 2 months). Mean ± SD, Student’s t-test, p<0.05 and p<0.01. (D) In serum from peripheral blood obtained as above, M-CSF levels were determined by ELISA using the Milliplex Kit (Millipore). Mean ± SD. Student’s t-test. (TIF) [file pone.0072674.s004.tif]
