# Supplementary material for: PI3K p110γ Deletion Attenuates Murine Atherosclerosis by Reducing Macrophage Proliferation but Not Polarization or Apoptosis in Lesions
Source: PLoS One. 2013 Aug 22;8(8):e72674. doi: 10.1371/journal.pone.0072674 (PMC3750002; doi:10.1371/journal.pone.0072674)
Supplement: Table S1 — Foxp3+ regulatory T cells infiltrate in atherosclerotic lesions from LDLR−/−p110γ+/− and LDLR-/−p110γ−/− mice. Quantitative analysis of Foxp3+ cells per aortic sinus section of indicated mice (n = 8/genotype). Results show mean ± SD. (DOC) [file pone.0072674.s005.doc]

**Table S1. Foxp3^+^ regulatory T cells infiltrate in atherosclerotic lesions from LDLR^-/-^p110γ^+/-^ and LDLR^‑/-^p110γ^-/-^** mice

| **nº Foxp3^+^ cells/section**  **(*n* = 3 sections/mouse)** | **LDLR^-/-^ p110γ^+/-^**  **(mean ± SD)** | **LDLR^-/-^ p110γ^-/-^**  **(mean ± SD)** |
| --- | --- | --- |
| Mouse 1 | 0 ± 0 | 0 ± 0 |
| Mouse 2 | 0.667 ± 0.578 | 0 ± 0 |
| Mouse 3 | 0.333 ± 0.578 | 0 ± 0 |
| Mouse 4 | 0 ± 0 | 0 ± 0 |
| Mouse 5 | 0 ± 0 | 0 ± 0 |
| Mouse 6 | 0.333 ± 0.578 | 0 ± 0 |
| Mouse 7 | 2.667 ± 0.578 | 0 ± 0 |
| Mouse 8 | 0.667 ± 0.578 | 0 ± 0 |
| **Total all mice** | **0.583 ± 0.886** | **0 ± 0** |

Quantitative analysis of Foxp3^+^ cells per aortic sinus section of indicated mice (*n*= 8/genotype). Results show mean ± SD.
