# Supplementary material for: PI3K p110γ Deletion Attenuates Murine Atherosclerosis by Reducing Macrophage Proliferation but Not Polarization or Apoptosis in Lesions
Source: PLoS One. 2013 Aug 22;8(8):e72674. doi: 10.1371/journal.pone.0072674 (PMC3750002; doi:10.1371/journal.pone.0072674)
Supplement: Supplement S1 — Supporting Materials and Methods, Results and References. (DOC) [file pone.0072674.s006.doc]

**SUPPLEMENT S1**

**Supporting materials and methods**

**Mouse experiments**

We conducted five independent mouse experiments (induction of atherosclerosis by feeding mice a high-fat diet for two months), the first to set up the system and the analyses, and four to obtain data. In each group, some mice were euthanized during the course of the experiment to monitor disease progression. Samples from at least two independent mouse experiments were used for each analysis.

**Analysis of serum lipid levels**

To analyze serum lipid levels, we anesthetized mice by intraperitoneal (i.p.) injection of ketamine (121 mg/kg) and xylazine (14 mg/kg); tail- and toe-pinch reflexes were tested to monitor adequacy of anesthesia. Whole blood was then extracted by retro-orbital bleeding before (t = 0) and after (t = 2 months) high-fat diet treatment, allowed to coagulate (room temperature (RT), 3 to 5 h) and centrifuged to collect serum, which was frozen (-20ºC). Samples were sent to an external laboratory (Labipath, Madrid, Spain) for analysis of lipid levels (total cholesterol, HDL-cholesterol, LDL-cholesterol and triglycerides). The CNB Ethics Committee for Animal Experimentation approved all animal studies (ref: 11021), in compliance with national and European Union legislation (Directive 2010/63/EU).

**Lesion area quantification**

Paraffin-embedded sections of the aortic valve region of LDLR^-/-^p110γ^+/-^ and LDLR^-/-^p110γ^-/-^ mice were hematoxylin/eosin stained. Serial sections were dewaxed in xylene and rehydrated in ethanol, incubated in Mayer’s hematoxylin (Sigma-Aldrich, St. Louis, MO; 15 min) and washed in water (10 min). Sections were incubated in eosin (2 to 4 min), washed twice in 70% ethanol, dehydrated with 100% ethanol and xylene, mounted in Entellan (Merck, Madrid, Spain) and dried at 37ºC. Aortic lesion area was quantified with ImageJ software.

**Immunohistochemical detection of CD3^+^ T cells, Mac-3^+^ macrophages and Foxp3^+^ regulatory T cells**

Hearts from LDLR^-/-^p110γ^+/-^ and LDLR^-/-^p110γ^-/-^ mice were fixed and paraffin-embedded. Serial sections were dewaxed and rehydrated as above. Antigens were exposed by steaming in sodium citrate buffer (15 min). Samples were blocked with peroxidase (Peroxidase Block, Dako, Glostrup, Denmark) and incubated (RT, 1 h) with the following primary antibodies: monoclonal rat anti-mouse Lamp-2 (Mac-3) (M3/84; Santa Cruz Biotech, Santa Cruz, CA), rat anti-mouse Foxp3-FITC (eBioscience, San Diego, CA) and rabbit anti-human CD3 (Dako). After several washes in 0.3% TBS-Triton, tissue was incubated with EnVision System-HRP (AEC) anti-rabbit for mouse tissues (Dako) and Simple Stain Rat MAX PO anti-rat for mouse tissues (Nichirei Biosciences, Tokyo, Japan) (RT, 1 h). The signal was developed with AEC (3-amino 9-ethylcarbazole) Substrate Chromogen (Dako; 15 min). Samples were hematoxylin counterstained and sections mounted in aqueous medium (Fluoromount-G, Southern Biotech, Birmingham, AL). Images were acquired using a Leica microscope (vertical Leitz DM RB) with an adapted Olympus (DP70) camera. Mac-3-stained area and CD3^+^ or Foxp3^+^ cell number in lesions were quantified with ImageJ by measuring Mac-3^+^ area per section or relative to total lesion area, or counting the number of total CD3^+^ or Foxp3^+^ cells in lesions per aortic sinus section, or relative to total lesion area.

***In vivo* determination of macrophage and T cell proliferation**

Slides were incubated (65ºC, 30 min) to improve section adhesion before dewaxing and rehydration. Antigens were exposed in sodium citrate buffer. Pap Pen (Research Products International, Mount Prospect, IL) was used to delimit sections. Samples were blocked with 1% bovine serum albumin (BSA, Sigma) in PBS (RT, 45 min). Primary antibodies (rat anti‑mouse Ki67 and rabbit anti-human CD3 (both from Dako), rat anti-mouse Lamp-2 (Santa Cruz), rabbit anti-mouse Ki67 (Master Diagnostica, Granada, Spain)) were diluted in blocking solution. After several washes in PBS, samples were incubated (RT, 45 min in a humidified chamber) with appropriate secondary antibody (Alexa647- or Alexa546-goat anti-rat or Alexa488-goat anti-rabbit (Molecular Probes/Invitrogen, Carlsbad, CA)) diluted in blocking solution. Nuclei were DAPI-stained (15 min), washed in PBS, and slides mounted in Fluoromount-G (Southern Biotech) or Slow Fade Gold Antifade Reagent (Invitrogen, Grand Island, NY). Images were acquired in a Zeiss LSM-510 META ConfoCor2 microscope and analyzed using LSM-FCS software and ImageJ. Macrophage and T cell proliferation was measured as the percentage of proliferating macrophages (Mac-3^+^Ki67^+^) or T cells (CD3^+^Ki67^+^) relative to total macrophages (Mac-3^+^) or T cells (CD3^+^) in plaques.

***In vivo* study of M1 and M2 macrophages**

M1 and M2 macrophages were analyzed by immunofluorescent staining of the aortic valve region in paraffin-embedded sections as for T cell and macrophage proliferation, but included incubation with purified anti-mouse IgG1κ antibody (eBioscience) prior to blocking. Primary antibodies were rabbit anti-mouse iNOS (Upstate/Millipore, Billerica, MA), mouse anti-mouse arginase1 (BD Transduction Laboratories, Franklin Lakes, NJ) and rat anti-mouse Lamp-2. Secondary antibodies were Alexa546-goat anti-rat, Alexa488-goat anti-rabbit, or Cy5-goat anti-mouse IgG (Jackson Immunoresearch, West Grove, PA) diluted in blocking solution. Quantification was performed with ImageJ.

***In vivo* determination of lesion apoptosis and of vascular smooth muscle cells**

Slides were incubated (65ºC, 30 min) to improve section adhesion before dewaxing and rehydration. Antigens were exposed in sodium citrate buffer. Pap Pen was used to delimit sections. Samples were blocked with 3% goat serum in PBS 0.25% Triton X-100 (RT, 45 min). Primary antibodies (anti-alpha smooth muscle actin (Abcam, Cambridge, UK), rabbit anti-mouse cleaved caspase-3 (Cell Signaling, Danvers, MA) and rat anti-mouse Lamp‑2 (Santa Cruz)) were diluted in blocking solution and incubated (4ºC, overnight). After several washes in PBS, samples were incubated (RT, 45 min in a humidified chamber) with appropriate secondary antibody (Alexa546-goat anti-rat or Alexa488-goat anti-rabbit (Molecular Probes/Invitrogen)) diluted in blocking solution. Nuclei were DAPI-stained (15 min), washed in PBS, and slides mounted in Fluoromount-G (Southern Biotech). Images were acquired in a Leica SP5 microscope and analyzed with ImageJ.

For TUNEL staining, slides were incubated (65ºC, 30 min) to improve section adhesion before dewaxing and rehydration, then washed several times with distilled water or PBS and permeabilized (PBS 0.5% Triton X-100, RT, 10 min). Slides were pre-incubated (RT, 15 min) with TdT1X buffer + 1 mM CoCl_2_ (TdT1X buffer: 125 mM Tris-HCl, 1.25 mg/ml BSA, 1 M sodium cacodylate (last two from Sigma), pH 6.6), in the dark in humidified chambers (as for all further incubations). Slides were incubated with reaction mix (recombinant terminal transferase and biotin-16-dUTP (both from Roche) diluted in TdT1X buffer + 1 mM CoCl_2_ (37ºC, 1 h), then washed with TdT1X buffer + 1 mM CoCl_2_ (RT, 10 min). The reaction was terminated with PBS + 0.01% Tween20, slides were washed, and incubated with streptavidin-Cy5 (Jackson) (RT, 1 h). Nuclei were DAPI-stained (RT, 15 min) and mounted with Fluoromount-G. Images were taken with a Leica SP5 confocal microscope and analyzed with Image J.

**Macrophage isolation and culture**

Mice were euthanized with CO_2_. Bone marrow-derived macrophages (BMM) were obtained from femoral bone marrow suspensions and allowed to differentiate for 7 days in Dulbecco’s Modified Eagle Medium (DMEM) supplemented with antibiotics, 20% fetal bovine serum (FBS) and 3 ng/ml recombinant M-CSF (Peprotech, Rocky Hill, NJ).

**Macrophage cell cycle analysis**

BMM were synchronized in G0/G1 by 18-36 h of M-CSF deprivation (DMEM, 10% FBS, 0.5% L929-cell-conditioned medium) and then stimulated for different times with DMEM, 10% FBS, 10% L929-cell-conditioned medium as a source of M-CSF or with DMEM, 10% FBS and 10 ng/ml GM-CSF (Peprotech). Macrophages were trypsinized and collected. After fixing in 80% ethanol (-20ºC, 1 h), cells were incubated at least 30 min with 50 μg/ml propidium iodide (P4170, Sigma) containing 0.25 mg/ml RNase A (R4642, Sigma). Labeled cells were analyzed in a FACSCanto flow cytometer (Becton Dickinson) and DNA histograms were fitted to cell cycle distributions using MultiCycle AV software (Phoenix Flow Systems, San Diego, CA).

**Analysis of intracellular cyclic adenosine monophosphate (cAMP) concentration**

For ELISA experiments, BMM were obtained from LDLR^-/-^p110γ^+/-^ and LDLR^-/-^p110γ^-/-^ mice as above. At 7 days post-differentiation, cells were arrested (18 h) by M-CSF deprivation, removed from Petri dishes and counted. Cell Lysis Buffer 5 from the Parameter Cyclic AMP Assay kit (KGE002B, R&D Systems) was added to each sample to a final concentration of 10^7^ cells/ml. Cell lysates were frozen (-80ºC); ELISA was performed according to manufacturer’s instructions.

For Western blot analysis, BMM from LDLR^-/-^p110γ^+/-^ and LDLR^-/-^p110γ ^-/-^ mice were *in vitro* differentiated and M-CSF-stimulated at several times (0, 24 and 48 h). Cells were washed in PBS, lysed in lysis buffer (25 mM Tris-HCl, pH 7.5, 1% (w/v) Triton X-100, 137 mM NaCl and 5% glycerol, supplemented with phosphatase and protease inhibitors (Sigma, St. Louis, MO)). Protein was quantified with the Micro BCA Protein Assay Kit (Thermo Scientific, Waltham, MA). Samples were analyzed in 12% SDS-PAGE for cAMP detection and 10% for p-CREB and CREB detection. cAMP, phospho-CREB (p-CREB) and CREB levels were detected in Western blot with anti-cAMP (clone SPM486, Abcam, Cambridge, UK), ‑p‑CREB (Ser133) and -CREB (clone 48H2; both from Cell Signaling, Danvers, MA). The signal was developed with ECL (Amersham, Barcelona, Spain). β-actin was used as loading control for cAMP Western blot (clone AC-15, Sigma); band intensity was quantified using ImageJ. The anti-cAMP antibody (SPM486) was generated using cAMP compounds as immunogen, and a chemically linked cAMP-carrier protein for antibody screening. In Western blot, this antibody recognizes protein-bound cAMP; in macrophages, it recognized three major bands of 24, 33 and 35 kDa, whose intensity was quantified with ImageJ. As a control, BMM from p110γ^+/-^ mice were *in vitro* differentiated and forskolin (FSK)-stimulated (100 μM, Sigma) at several times (0, 24 and 48 h). cAMP was detected by Western blot and β-actin was used as loading control.

**Flow cytometry staining to study peripheral blood immune cell populations**

Peripheral blood was extracted from LDLR^-/-^p110γ^+/-^ and LDLR^-/-^p110γ^-/-^ mice and held in EDTA-treated tubes (RT) until staining. Blood (20-30 μl) was transferred to flow cytometry tubes; 50 μl of antibody mix (anti-mouse CD45-PE (Southern Biotech), -mouse CD11b-biotin, -mouse Ly6C-FITC, -mouse CD3-FITC (all from Beckman Coulter, Indianapolis, IN) and -mouse Gr1-PerCPCy5.5 (BD Pharmingen, San Diego, CA)) was added to each tube and incubated (RT, 20 min). Where biotinylated antibodies were used, 500 μl PBS were added (RT) and centrifuged (300 x g, 15 min). Supernatant was discarded and 50 μl avidin-EF450 (eBioscience) added and incubated (RT, 20 min, in the dark); 1 ml Versalyse in 0.1% PFA was added to each tube and mixed by vortexing (1-3 sec). Tubes were incubated (RT, 10 min, in the dark) and samples analyzed in a Gallios Flow Cytometer (Beckman Coulter).

**Serum M-CSF concentration in LDLR^-/-^p110γ^+/-^ and LDLR^-/-^p110γ^-/-^ mice**

Serum was obtained from LDLR^-/-^p110γ^+/-^ and LDLR^-/-^p110γ^-/-^ mice as described for lipid level determination before (t = 0) and after (t = 2 months) high-fat diet treatment. M-CSF levels were quantified with a Milliplex Kit (Millipore) in a Luminex 100 IS (Luminex Corporation, Austin, TX).

**qRT-PCR analysis of M1 and M2 macrophage marker expression**

RNA was extracted with TRI Reagent (Sigma) from *in vitro*-polarized M1 and M2 macrophages from p110γ^+/-^ and p110γ^-/-^ mice. cDNA was obtained from 1 μg total RNA using the High Capacity cDNA Reverse Transcription Kit 496 thermocycler (MWG AG Biotech, Ebersberg, Germany). Several dilutions of each cDNA were used as templates for PCR amplification. Oligonucleotide primers used were iNOS, IL-12, arginase1, IL-10 and YM1. For iNOS, forward 5’-GCTGTGCTCCATAGTTTCCAG-3’, reverse 5’-GGACCAGCCAAA TCCAGTC-3’; for IL-12, forward 5’-TGGTTTGCCATCGTTTTGCTG-3’, reverse 5’-ACAGGTGAGGTTCACTGTTTCT-3’; for arginase 1, forward 5’‑TCCAAGCCAAAGTCC TTAGAG-3’, reverse 5’-AGGAGCTGTCATTAGGGACATC-3’; for IL-10, forward 5’-AGGCGCTGTCATCGATT TCTC-3’, reverse 5’-TGGCCTGTAGACACCTTGGTC‑3’; for YM1, forward 5’-ACTTTGATGGCCTCAACCTG-3’, reverse 5’-AATGATTCCTGCTCCT GTGG-3’; for β-actin, forward 5’-GGCTCCTAGCACCATGAAGA-3’, reverse 5’-CCACCG ATCCACACAGAGTA-3’. Amplification was performed at 95ºC, 10 min; 40 cycles, 95ºC, 15 sec; 60ºC, 20 sec; 72ºC, 30 sec. To confirm amplification purity, a dissociation curve was generated at 95ºC, 15 sec; 60ºC, 15 sec and 2% ramp to 95ºC. mRNA levels were quantified using the 2^-ΔCt^ method and normalized to an endogenous reference (β‑actin) and relative to a calibrator. Results were analyzed using SDS v2.2.2.

**Supporting results**

**Analysis of serum lipid levels and lesion area quantification**

Serum levels of total, HDL- and LDL-cholesterol, and triglycerides were measured pre- and post-diet. High-fat diet increased serum cholesterol, LDL-cholesterol and triglycerides similarly in LDLR^-/-^p110γ^+/-^ and LDLR^-/-^p110γ^-/-^ mice at all times tested (Figure S1A). We analyzed atherosclerosis burden by planimetric analysis of hematoxylin/eosin-stained cross-sections of the aortic sinus (Figure S1B). Lesion size (mm^2^) was smaller in LDLR^-/-^p110γ^-/-^ than in LDLR^-/-^p110γ^+/-^ mice (Figure S1C).

**Study of circulating immune cell populations in LDLR^-/-^p110γ^+/-^ and LDLR^-/-^p110γ^-/-^ mice**

p110γ^+/-^ and p110γ^-/-^ mice show no differences in circulating immune cell populations except for neutrophils, which are more abundant in p110γ^-/-^ than in p110γ^+/-^ mice [1]. To ensure that peripheral blood immune cell populations were unaltered in LDLR^-/-^p110γ^-/-^ compared to LDLR^‑/-^p110γ^+/-^ mice, we analyzed the percentage of T cells (CD3^+^), inflammatory monocytes (Ly6C^+^) and granulocytes (Gr1^+^) in peripheral blood from LDLR^-/-^p110γ^+/-^ and LDLR^-/-^p110γ^-/-^ mice before and after two months on a high fat diet (Figure S4A-C). To determine whether lack of p110γ PI3K affected M-CSF serum levels, we measured serum M-CSF concentration in LDLR^-/-^p110γ^+/-^ and LDLR^-/-^p110γ^-/-^ mice before and after two months on a high-fat diet (Figure S4D). There were no differences in the percentage of inflammatory monocytes or T cells between LDLR^-/-^p110γ^+/-^ and LDLR^-/-^p110γ^-/-^ mice at the two times analyzed (Figure S4A, B), whereas the percentage of blood neutrophils was higher in LDLR^-/-^p110γ^-/-^ than in LDLR^-/-^p110γ^+/-^ mice at both times (Figure S4C). Serum M-CSF concentration in peripheral blood did not differ between the mice at t = 0 or t = 2 months on a high-fat diet (Figure S4D).

**Supporting References**

1. Hirsch E, Katanaev VL, Garlanda C, Azzolino O, Pirola L, et al. (2000) Central role for G protein-coupled phosphoinositide 3-kinase gamma in inflammation. Science 287: 1049-1053.
